# Effects of storage conditions on the stability of qPCR reagents: implications for environmental DNA detection

**DOI:** 10.1186/s13104-024-06850-4

**Published:** 2024-07-18

**Authors:** Mark Louie D. Lopez, Ellika M. Crichton, Michael J. Allison, Anna H. Dema, Matthew T. Bonderud, Caren C. Helbing

**Affiliations:** 1 Department of Biochemistry & Microbiology, University of Victoria, Victoria, BC V8P 5C2 Canada; 2 Present Address: Pacific Biological Station, Fisheries and Oceans Canada, Nanaimo, BC V9T 6N7 Canada

**Keywords:** eDNA, qPCR master mix, Freeze–thaw cycles, Primer and probe stability, Synthetic DNA

## Abstract

**Objective:**

Environmental DNA (eDNA) detection is a transformative tool for ecological surveys which in many cases offers greater accuracy and cost-effectiveness for tracking low-density, cryptic species compared to conventional methods. For the use of targeted quantitative PCR (qPCR)-based eDNA detection, protocols typically require freshly prepared reagents for each sample, necessitating systematic evaluation of reagent stability within the functional context of eDNA standard curve preparation and environmental sample evaluation. Herein, we assessed the effects of long-term storage and freeze–thaw cycles on qPCR reagents for eDNA analysis across six assays.

**Results:**

Results demonstrate qPCR plates (containing pre-made PCR mix, primer-probe, and DNA template) remain stable at 4 °C for three days before thermocycling without fidelity loss irrespective of qPCR assay used. Primer-probe mixes remain stable for five months of − 20 °C storage with monthly freeze-thaw cycles also irrespective of qPCR assay used. Synthetic DNA stocks maintain consistency in standard curves and sensitivity for three months under the same conditions. These findings enhance our comprehension of qPCR reagent stability, facilitating streamlined eDNA workflows by minimizing repetitive reagent preparations.

**Supplementary Information:**

The online version contains supplementary material available at 10.1186/s13104-024-06850-4.

## Introduction

Environmental DNA (eDNA) detection has emerged as a sensitive and cost-effective tool for detecting rare species, outperforming conventional survey methods. It requires the isolation of genetic materials from environmental samples such as water, soil, and air to detect the presence/absence of target species [1, 2]. Despite its potential, variable data quality impedes general use and regulatory integration, necessitating measures to eliminate false positives and negatives [3, 4].

In the past years, standardized frameworks have been developed for eDNA workflows, particularly in targeted quantitative (qPCR)-based detection [5, 6]. This method detects specific genomic regions using primer pairs and probes typically using freshly prepared reagents. However, the stability of these reagents in eDNA analyses remains understudied, despite the significant impact of storage conditions, especially temperature, on their longevity. While qPCR reagents and synthetic DNA templates are stable at − 20 °C for up to 24 months, repeated freeze–thaw cycles may affect their quality due to UV and ambient light exposure, higher temperatures, and contamination [7, 8]. Aliquoting reagents in lesser amounts can help to attenuate these effects [9].

Despite these insights, the influence of storage conditions on the stability of various qPCR reagents and, consequently, the effectiveness of eDNA detections across multiple eDNA assays is poorly understood. Herein, we systematically examined the following: (1) qPCR reaction mix stability in the detection of low and high DNA copies in the context of prepared PCR plates; (2) the effect of long-term storage and repeated thawing on primer–probe mixes; and (3) the stability of gBlocks® synthetic DNA templates and its effect on standard curves. The present study provides a valuable framework and guidance for confidently streamlining routine handling and storage of reagents for qPCR-based eDNA studies.

## Methods

### eDNA assay design and validation

Six previously validated eDNA qPCR-based assays were used in the present study: eAMPE5 (*Ammodytes personatus* [10]); eCACO4 (*Catostomus commersonii* [11]), eCOCL1 (*Coregonus clupeaformis* [12]), eFISH1 (general fish DNA [12]), eLIPI1 (*Lithobates* (*Rana*) *pipiens* [13]); and eONMY5 (*Oncorhynchus mykiss* [11]) (Additional Table 1). Each assay was developed and validated as described previously [14, 15] and performance characteristics were consistent with the Canadian standard on performance criteria for the analyses of eDNA by targeted qPCR [5, 6].

### Experiment 1: qPCR reaction mix stability testing in the context of prepared plates

To evaluate how short-term storage affected the stability of prepared reaction plates with qPCR master mix, DNA copy number was determined from two reconstituted gBlocks® of the appropriate DNA sequence (4 and 20 copies/reaction) using three different assays (eAMPE5, eFish1, and eLIPI1; Additional Figure 1). To increase the likelihood of detecting the target amplicon, samples were analyzed using eight technical replicates per assay [16]. Eight additional replicates of non-template control (NTC) using UltraPure-dH_2_O (Invitrogen, Massachusetts, USA) were also run. Two identical sets of qPCR plates were made; one set was immediately run for qPCR analysis, while the other plate was stored at 4 °C for three days before qPCR analysis.

Each qPCR reaction consisted of 2 µL of known copies of diluted gBlocks® template, 700 nM forward and reverse primers, 100 nM TaqMan probe, and 1X of QIAcuity Probe Master Mix (QIAcuity Probe PCR Kit, QIAGEN, Hilden, Germany) for a final reaction volume of 15 µL. The following TaqMan thermocycler profile was used for all assays: initial denaturation of 9 min at 95 °C followed by 50 cycles of 15 s at 95 °C, 30 s at 64 °C and 30 s at 72 °C. The eDNA concentration (copies/reaction) of amplified samples was extrapolated from C_q_ values using the previously generated standard curves [16].

### Experiment 2: qPCR primer–probe mix stability testing

Three fish eDNA assays (eCACO4, eCOCL1, and eFISH1) were selected for the qPCR primer–probe mix stability test (Additional Figure 1). Freshly made primer–probe mixes for each assay containing 7 µM each of forward and reverse primers and 1 µM TaqMan probe were aliquoted and designated to a freeze–thaw treatment (with and without monthly thawing) and time for running qPCR plates (months 0 to 5). For the duration of the experiment, the primer–probe aliquots were kept at − 20 °C in a manual defrost freezer and protected from light. Each month the designated primer–probe aliquots were thawed, used to make the master mixes, then returned to − 20 °C or discarded depending on the treatment condition. Each qPCR primer–probe mix treatment received eight technical replicates of 2 µL reconstituted gBlocks® (@10 copies/µL = 20 copies/reaction) and two technical NTC replicates of UltraPure-dH_2_O (Invitrogen, Massachusetts, USA). The results were then converted to copies/L. This experiment was repeated every month for a total of five months.

### Experiment 3: Synthetic DNA template (gBlocks®) stability testing

To investigate the effect of storage time on the stability of the gBlocks® template, four different eDNA assays were selected: eAMPE5, eFish1, eLIPI1, and eONMY5 (Additional Figure 1). For each corresponding assay, new gBlocks® dilution series were made in the following concentrations: 10^10^, 10^8^, 10^7^, 321,500, 32,150, 6250, 1250, 250, 50, 10, 2, 4, 0.8, and 0.016 copies per µL. All lyophilized gBlocks® were suspended in tRNA (10 ng/µL; Sigma-Aldrich, Missouri, USA) as a stabilizer in all DNA standard solutions, except for the initial stock. To reduce the impacts of freeze–thaw cycles, four aliquots (60 µL) of the 32,150 to 0.016 (copies/reaction) dilutions were made to match different periods for running the qPCR plates (months 0 to 3). For the duration of the experiment, the aliquots of reconstituted gBlocks® were kept at − 20 °C in a manual defrost freezer. For each 15 µL reaction, 2 µL of synthetic DNA solution was added. Identical qPCR plates were prepared for each assay at each time.

The sensitivity of gBlocks® dilutions was assessed by comparing the derived limits of detection (LOD) and limits of quantification (LOQ) that were calculated using eLowQuant, which is based on a modified Binomial-Poisson distribution model using the generated standard curve [16]. These values indicate the lowest DNA concentration that may be determined with adequate statistical certainty using eDNA tests. The LOD from continuous data (LOD_continuous_) was also determined as the lowest copy number at which there is a ≥ 95% detection [6, 13]. The LOD_continuous_ indicates the breakpoint for continuous and discontinuous data defining the computational approaches for determining sample copy number [6]. Lastly, the linear regression equation for each assay was also checked to calculate the PCR assay efficiency for each constructed eDNA assay, which measures the capacity of the specified primers and probe to amplify the target DNA region for every qPCR cycle. Reagents used in all experiments were placed in low adsorption boil proof Corning® Eppendorf tubes (MilliporeSigma, Darmstadt, Germany).

### Statistical analyses

R Studio (R Studio, Inc.) version 1.2.1335 was used to analyze the qPCR data. To reduce the effects of outlier measurements, data are presented as median values with median absolute deviation error. The Shapiro–Wilk test and Levene’s test were used to test the median copy-per-reaction values’ normality and homogeneity of variance, respectively. Non-parametric analyses were performed after it was determined that the prerequisites for normality and homogeneity had not been met. The Wilcoxon Signed Rank test was used to evaluate pairwise significance between DNA copy estimates for each month relative to the initial measurement (month 0). The Friedman repeated measures test was also used to determine if there were significant differences among test groups in Experiments 2 and 3.

## Results and discussion

### Experiment 1: qPCR reaction mix stability testing in the context of prepared plates

**Fig. 1:**
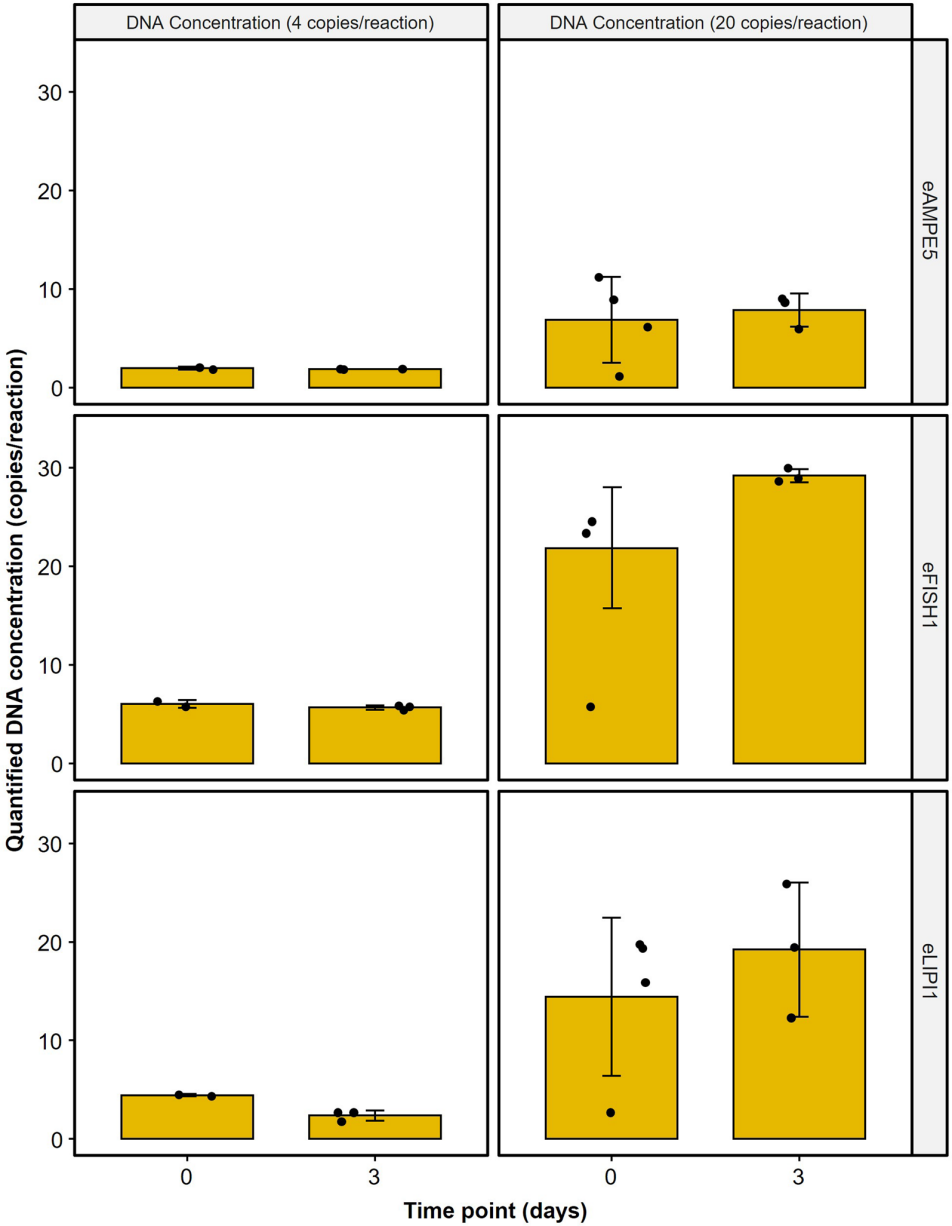
Median DNA concentration (n = 8) from target concentrations of **A** 4 copies/reaction or **B** 20 copies/reaction from three indicated eDNA assays that were run immediately (day 0) or the prepared plates stored at 4 °C and run three days later (day 3). All pairwise comparisons were not significantly different from each other. Full statistical results are presented in Additional Table 2

For eAMPE5, eFish1, and eLIPI1 assays, the qPCR master mixes with DNA template that were run at Days 0 and 3 resulted in comparable DNA copy estimates for gBlocks® dilutions of 4 or 20 copies per reaction (Additional Table 2). Pairwise analyses showed that storing prepared reaction plates containing the master mix and DNA template at 4 °C for three days had no significant effect on the estimated DNA copies (Wilcoxon; Fig. 1; p-value > 0.05) in comparison to the plate that was run on the same day of preparation (Day 0).

In laboratories handling numerous eDNA projects, simultaneous preparation of multiple qPCR plates is necessary due to equipment limitations. Frost-free freezers are not recommended as they undergo multiple freeze–thaw cycles and may therefore impact reagent integrity. To protect Taq polymerase from freezing conditions, glycerol is included in the storage buffer as a cryoprotectant [17]. We assessed multiple targeted eDNA assays, finding that storing prepared qPCR plates at 4 °C for up to three days does not affect DNA copy estimates, enabling batch preparation and storage, particularly beneficial for labs with limited resources. This allows for increased throughput of eDNA analyses, optimizing efficiency in high-throughput or resource-shared settings.

### Experiment 2: Testing the effect of qPCR primer–probe mix storage and handling conditions

**Fig. 2:**
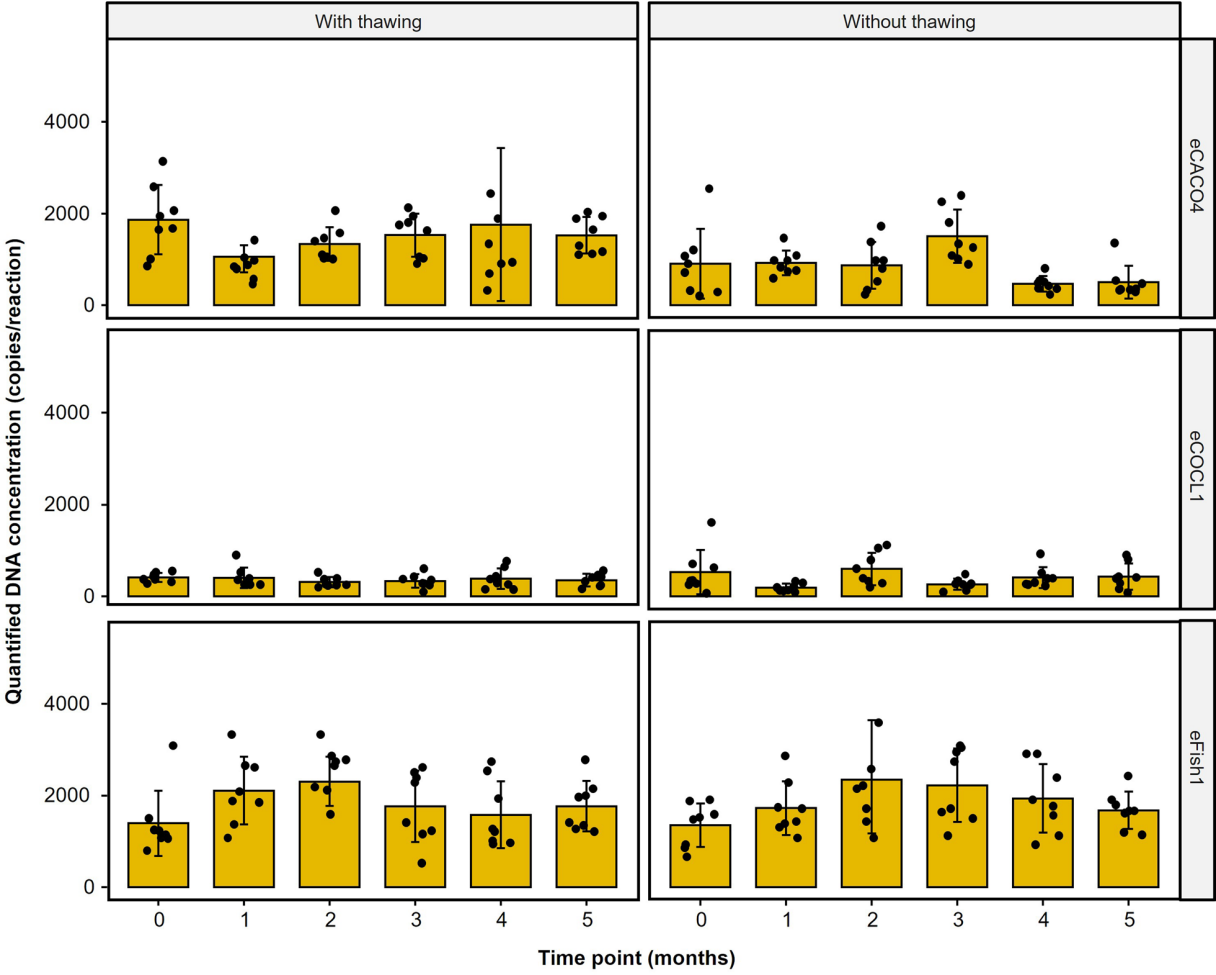
Median DNA concentration (n = 8) using primer–probe mixtures stored for five months. Two independent treatments were prepared to see the effect of a monthly freeze–thaw cycle (“With thawing”) compared to constant maintenance at − 20 °C (“Without thawing”). There were no significant differences between any time points. Full statistical results are presented in Additional Tables 3 and 4

The effects of repeated monthly freeze–thaw cycles of prepared primer–probe mixes up to 5 months did not have a significant impact on the downstream DNA quantification for the selected eDNA assays (Fig. 2; Additional Table 3). Pairwise analysis of the quantified DNA copies for each month showed no significant difference relative to the initial measurement (month 0; p-value > 0.05) for each eDNA assay. Moreover, the prolonged storage of the primer–probe mixture for up to 5 months had no significant effect on the quantified DNA copies (Fig. 2; Additional Table 4). Similarly, pairwise analysis of the DNA copy estimates for each month relative to the initial measurement (month 0) showed no significant differences (Wilcoxon; p-value > 0.05) for eCACO4, eCOCL1, and eFish1 eDNA assays. Overall, there were no significant differences observed in quantified DNA copies between two storage treatments (Friedman; p-value > 0.05).

Lyophilized primers remain stable for up to 25 weeks at 37 °C [18]. Probes, being sensitive to light and UV, require storage in amber tubes at − 20 °C to prevent degradation; they stay stable for about a year post-resuspension [18]. Freshly prepared primer–probe mixes are advised for each qPCR reaction to avoid freeze–thaw cycles and DNase contamination [19]. However, the present study indicates that proper handling, including protection from light exposure and DNase contamination, renders prolonged storage and freeze–thaw cycles insignificant for primer and probe stability.

### Experiment 3: Synthetic DNA template (gBlocks®) stability testing

The C_q_ values measured from the series of gBlocks® dilutions from 32,150 to 0.016 copies per µL were unaffected by continuous storage at − 20 °C or being subjected to monthly freeze–thaw cycles. Several sensitivity parameters such as amplification efficiency, coefficient of correlation (R^2^), LOD, LOQ, and LOQ_continuous_ for the four different qPCR-based eDNA assays (eAMPE5, eFish1, eLIPI1, and eONMY5) remained constant over three months (Table 1, Additional Tables 5–8). Overall, there were no significant differences observed in quantified DNA copies among different time points (Friedman; p-value > 0.05).

**Table 1:** Summary of assay sensitivity parameters-based qPCR runs on different time points using known synthetic DNA template (gBlocks®) concentrations

eDNA assays	Time point (months)	Amplification efficiency (%)	R^2^	LOD ± 95% CI (copies/L)	LOQ ± 95% CI (copies/L)	LOD_continuous_ (copies/L)
eAMPE5	1	94	1	0.3 ± 0.3	1.3 ± 0.8	4
	2	105	1	0.3 ± 0.3	1.2 ± 0.9	4
	3	97	0.998	0.3 ± 0.3	1.2 ± 1.0	4
eFish1	0	107	0.999	0.2 ± 0.2	0.8 ± 0.7	4
	1	111	0.998	0.4 ± 0.3	1.5 ± 0.7	4
	2	117	0.994	0.2 ± 0.1	0.7 ± 0.4	4
	3	111	0.998	0.2 ± 0.1	0.7 ± 0.5	4
eLIPI1	0	107	0.996	0.2 ± 0.2	0.9 ± 0.6	4
	1	99	1	0.2 ± 0.2	0.9 ± 0.7	4
	2	107	0.998	0.2 ± 0.2	0.8 ± 0.6	4
	3	102	1	0.5 ± 0.2	1.0 ± 0.7	4
eONMY5	0	103	1	0.4 ± 03	1.4 ± 1.3	4
	1	89	0.998	0.3 ± 0.3	1.2 ± 0.9	4
	2	103	0.999	0.4 ± 0.3	1.4 ± 1.4	4
	3	99	0.999	0.3 ± 0.2	1.1 ± 0.8	4

*CI* confidence interval

Many eDNA labs utilize resuspended gBlocks® dilutions for constructing standard curves to quantify DNA copies in environmental samples [5]. Stabilizing standard stocks with TE buffer (pH 8) and tRNA helps protect against DNases and hydrolysis [20]. Including stabilizers in all ds synthetic DNA standard solutions, except the initial high-concentration stock, is recommended [21]. The use of tRNA in standard dilutions prevents low-copy standards from losing amplifiable DNA due to nonspecific adsorption on vial plastic over time [13]. While it is common practice to use freshly made standard solutions in each qPCR run, our study found that standard stocks exposed to freeze–thaw cycles had no significant impact on sensitivity features. PCR amplification efficiency, LOD, and LOQ remained highly comparable across all time points. Thus, gBlocks® standard stocks can be prepared in advance and stored at − 20 °C for three months without affecting standard curve accuracy for eDNA quantification via focused qPCR assays.

## Conclusions

Overall, the present study provides fundamental answers about the handling and storage of essential qPCR reagents used for targeted qPCR eDNA detection and a framework for testing other contexts for primer–probe mixes, thermostable DNA polymerases, and ds synthetic DNA. This information can increase confidence in the accuracy of the results from eDNA analysis. This allows researchers to streamline eDNA workflows to make them more time-efficient and eliminate extremely repetitive tasks needed in reagent preparations by providing information on the stability of primers, probes, and synthetic DNA templates being used in targeted qPCR-based eDNA analysis.

## Limitations

While we used multiple distinct targeted qPCR assays, we concentrated on evaluating the stability of qPCR reagents using one Taq DNA enzyme type, buffer mix, primer/probe concentrations, and synthetic double stranded DNA (gBlocks®) under specific, operationally relevant storage conditions. It is likely that the observations made herein are applicable to other Taq enzymes and reaction mixes. However, this should be empirically tested. It is also not known whether similar stability could be expected when using eDNA samples from environmental matrices.

### Supplementary Information

Supplementary Material 1.

## Data Availability

Raw data are available upon request to the corresponding author.

## Abbreviations

eDNA: Environmental deoxyribonucleic acid
qPCR: Quantitative real time polymerase chain reaction
ds synthetic DNA: Double strand synthetic ribonucleic acid
