# Supplementary material for: Effects of storage conditions on the stability of qPCR reagents: implications for environmental DNA detection
Source: BMC Res Notes. 2024 Jul 18;17:199. doi: 10.1186/s13104-024-06850-4 (PMC11264737; doi:10.1186/s13104-024-06850-4)
Supplement: Supplementary file 1 — Supplementary Material 1. [file 13104_2024_6850_MOESM1_ESM.pdf]

Supplementary Information

Effects of storage conditions on the stability of qPCR reagents: Implications for environmental DNA detection

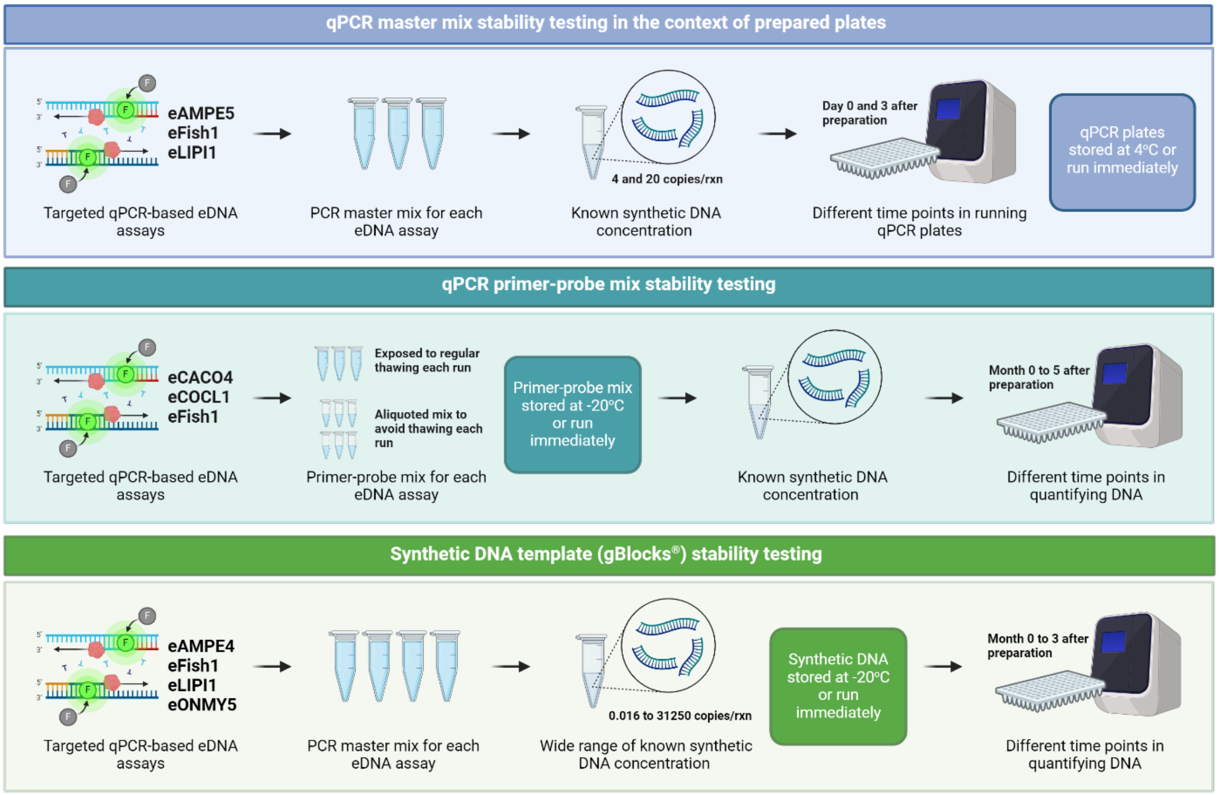

Additional figure 1. Experimental designs for testing qPCR master mix, primer-probe mix, and synthetic DNA (gBlocks®) template stability. The manipulated variables for each experiment are indicated in the colored boxes.

Additional table 1. Summary of primer and probe sequences for qPCR-based environmental DNA assay used in the present study. *mt-nd2*, mitochondrial NADH dehydrogenase 2; *mt-rnr1*, mitochondrial 12S ribosomal RNA

Assay	Target species	Common name	Sequence type	Sequence 5' -> 3'	Target gene	Amplicon size	Source
eAMPE5	*Ammodytes personatus*	Pacific sandlance	Forward	AGACGCCAAGACAGACC	*mt-rnr1*	164	Robinson et al., 2022
			Reverse	CTGTTCATTAGAGCTGTAGC			
			Probe	FAM-CCATAAGGAGCCCAAACCAAATGGT-ZEN/IB			
eCACO4	*Catostomus commersonii*	White sucker	Forward	CTAACTCTCCTTGCCCTA	*mt-nd2*	133	Lopez et al., 2023
			Reverse	TGGTAGTAACTATAATTGGACTT			
			Probe	FAM-TTCATAACATCAGCAGCATTCCTTACACT-ZEN/IB			
eCOCL1	*Coregonus clupeaformis*	Lake whitefish	Forward	CATCATTCCTCTCATAGCA	*mt-nd2*	162	Lopez et al., 2023
			Reverse	ATTGGGTGGGTTAATTGT			
			Probe	FAM-CCATTCTCCAACCAGTCAAGCATTAGT-ZEN/IB			
eFISH1	Fish DNA	General fish	Forward	CACCTAGAGGAGCCTGTTCTA	*mt-rnr1*	153	Klymus et. al., 2020
			Reverse	CTACACCTCGACCTGACGTT			
			Probe	FAM-TATATACCRCCGTCGTCAGCTTACCC-ZEN/IB			
eLIPI1	*Lithobates* (*Rana*) *pipiens*	Northern leopard frog	Forward	AGCTTACCATGTGAACGTCTT	*mt-rnr1*	1164	Klymus et. al., 2020
			Reverse	TACTACTAAATCCACCTTCGCT			
			Probe	FAM-CAATTGGCTACAATTTCTAATATAGAACAA-ZEN/IB			
eONMY5	*Oncorhynchus mykiss*	Rainbow trout	Forward	GTCTCTCCCTGTATATCG	*mt-nd2*	78	Lopez et al., 2024
			Reverse	GAGTATTGATGGTTAAAGAGT			
			Probe	FAM-TGACATCTTCAGCCTTCCTCACAT-ZEN/IB			

Additional table 2. Median C_q values and DNA copy number estimates for duplicate qPCR plates prepared on day 0 and either run immediately or stored at 4°C and run 3 days later. CI, confidence interval; rxn, reaction

Assay	DNA copy treatments (copies/rxn)	Time point (days)	Median C_q ± 95% CI	Median DNA (copies/rxn) ± 95% CI	P value (Wilcoxon signed rank test)
eAMPE5	4	0	36.37±0.21	1.97±0.10	0.50
		3	37.01±0.54	1.87±0.01	
	20	0	35.23±0.73	6.87±2.17	0.49
		3	34.29±0.17	7.87±0.97	
eFish1	4	0	35.51±0.98	6.00±0.28	0.41
		3	35.73±0.19	5.66±0.13	
	20	0	33.87±0.93	21.80±5.83	0.31
		3	32.30±0.27	29.17±6.38	
eLIPI1	4	0	34.20±0.09	4.45±0.09	0.39
		3	35.97±0.33	2.39±0.30	
	20	0	33.32±0.92	14.43±4.01	0.41
		3	32.11±0.90	19.21±3.93	

Additional table 3. Median C_q values and DNA copy number estimates for primer-probe mixture stored for up to five months exposed to monthly freeze-thaw cycles. CI, confidence interval

Assay	Time point (months)	Median C_q ± 95% CI	Median DNA (copies/L) ± 95% CI	P value (Wilcoxon signed rank test relative to 0 month time point)
eCACO4	0	31.03±0.29	1866.19±757.77	-
	1	31.24±0.23	1374.72±294.18	0.42
	2	31.22±0.17	1338.05±368.48	0.43
	3	31.14±0.22	1531.28±468.24	0.59
	4	31.24±0.28	1758.05±669.27	0.59
	5	31.13±0.18	1527.32±397.07	0.59
eCOCL1	0	32.19±0.19	415.22±97.87	-
	1	32.25±0.28	403.12±221.60	0.86
	2	32.39±0.21	310.69±109.64	0.71
	3	32.37±0.33	334.99±147.89	0.86
	4	32.31±0.38	386.23±223.30	0.86
	5	32.32±0.27	353.18±136.80	0.86
eFish1	0	32.64±0.26	1392.07±713.16	-
	1	32.35±0.24	2106.90±734.99	0.14
	2	32.31±0.15	2131.52±539.96	0.13
	3	32.51 ±0.37	1763.75±778.85	0.52
	4	32.56±0.29	1576.09±729.09	0.67
	5	32.46±0.20	1765.69±550.04	0.30

Additional table 4. Median C_q values and DNA copy number estimates for primer-probe mixture stored for up to five months with no freeze-thaw cycle exposure. CI, confidence interval

Assay	Time point (months)	Median C_q ± 95% CI	Median DNA (copies/L) ± 95% CI	P value (Wilcoxon signed rank test relative to 0 month time point)
eCACO4	0	31.66±0.20	907.61±269.40	-
	1	31.47±0.07	928.63±95.27	0.48
	2	31.61±0.16	869.86±181.34	0.91
	3	31.96±0.09	507.4±104.33	0.10
	4	32.07±0.13	415.11±72.14	0.35
	5	31.95±0.12	504.39±125.64	0.35
eCOCL1	0	32.23±0.21	530.83±170.66	-
	1	32.72±0.10	189.48±30.22	0.19
	2	32.04±0.14	599.72±125.46	0.59
	3	32.52±0.12	264.57±42.75	0.43
	4	32.24±0.29	410.72±228.66	0.95
	5	32.30±0.51	433.34±287.93	0.86
eFish1	0	32.65±0.26	1351.92±475.73	-
	1	32.48±0.21	1840.42±512.65	0.34
	2	32.28±0.32	2450.52±582.30	0.20
	3	32.32±0.26	2223.91±805.25	0.20
	4	32.42±0.22	1936.47 ±749.79	0.34
	5	32.48±0.06	1675.69±143.30	0.34

Additional table 5. Raw data and calculated sensitivity parameters for eAMPE5 sensitivity validation using synthetic gDNA (gBlocks®) dilutions. LL, lower limit; LOB, Limit of blank; LOD, limit of detection; LOQ, limit of quantification; $LOQ_{continuous}$, continuous LOQ; UL, upper limit; rxn, reaction

Month	Total Copies/rxn	Hit Frequency	Average C_q	Standard Error	Efficiency	R^2	LOD LL (copies/rxn)	LOD (copies/rxn)	LOD UL (copies/rxn)	LOQ LL (copies/rxn)	LOQ (copies/rxn)	LOQ UL (copies/rxn)	$LOQ_{continuous}$ (copies/rxn)	LOB
0	62500	8/8	21.89	0.06	95%	0.9956	0.2	0.3	0.5	0.8	1.1	1.9	4	0
	12500	8/8	24.24	0.06										
	2500	8/8	26.52	0.08										
	500	8/8	28.96	0.06										
	100	24/24	31.99	0.23										
	20	24/24	33.30	0.24										
	4	24/24	36.56	0.19										
	0.8	16/24	38.34	0.16										
	0.16	4/24	37.77	0.14										
	0.032	0/24	0.00	0.00										
	0	0/24	0.00	0.00										
1	62500	8/8	22.10	7.81	94%	0.9995	0.2	0.3	0.6	0.9	1.3	2.4	4	0
	12500	8/8	24.33	8.60										
	2500	8/8	26.73	9.45										
	500	8/8	29.08	10.28										
	100	24/24	31.50	6.43										
	20	24/24	34.14	6.97										
	4	24/24	36.59	7.47										
	0.8	13/24	38.55	7.87										
	0.16	5/24	38.64	7.89										
	0.032	1/24	39.04	7.97										

	0	0/24	0.00	0.00										
2	62500	8/8	22.76	0.03	105%	0.998	0.2	0.3	0.6	0.8	1.2	2.1	4	0
	12500	8/8	25.16	0.03										
	2500	8/8	27.59	0.04										
	500	8/8	30.05	0.04										
	100	24/24	32.13	0.09										
	20	24/24	33.99	0.09										
	4	24/24	36.28	0.20										
	0.8	12/24	38.66	0.38										
	0.16	8/24	39.17	0.20										
	0.032	1/24	40.18	#DIV/0!										
	0	0/24	#DIV/0!	#DIV/0!										
3	62500	8/8	22.00	0.13	97%	0.9983	0.2	0.3	0.6	0.8	1.2	2.2	4	0
	12500	8/8	24.29	0.15										
	2500	8/8	26.34	0.08										
	500	8/8	28.69	0.20										
	100	24/24	31.13	0.40										
	20	24/24	33.71	0.45										
	4	24/24	36.34	0.77										
	0.8	17/24	38.60	3.06										
	0.16	1/24	36.71	#DIV/0!										
	0.032	1/24	36.50	#DIV/0!										
	0	0/24	#DIV/0!	#DIV/0!										

Additional table 6. Raw data and calculated sensitivity parameters for eFish1 sensitivity validation using synthetic gDNA (gBlocks®) dilutions. LL, lower limit; LOB, Limit of blank; LOD, limit of detection; LOQ, limit of quantification; $LOQ_{continuous}$, continuous LOQ; UL, upper limit; rxn, reaction

Month	Total Copies/rxn	Frequency	Average	Standard Error	Efficiency	R^2	LOD LL (copies/rxn)	LOD (copies/rxn)	LOD UL (copies/rxn)	LOQ LL (copies/rxn)	LOQ (copies/rxn)	LOQ UL (copies/rxn)	$LOQ_{continuous}$ (copies/rxn)	LOB
0	62500	8/8	20.23	0.04	95%	0.9956	0.2	0.3	0.5	0.8	1.1	1.9	4	0
	12500	8/8	22.47	0.05										
	2500	8/8	24.75	0.05										
	500	8/8	27.09	0.04										
	100	24/24	29.52	0.06										
	20	24/24	31.25	0.05										
	4	24/24	33.47	0.13										
	0.8	17/24	35.39	0.21										
	0.16	7/24	36.60	0.12										
	0.032	1/24	37.29	<0.01										
	0	0/24	0.00	0.00										
1	62500	8/8	20.40	0.04	94%	0.9995	0.2	0.3	0.6	0.9	1.3	2.4	4	0
	12500	8/8	22.67	0.05										
	2500	8/8	24.86	0.05										
	500	8/8	27.21	0.02										
	100	24/24	29.35	0.06										
	20	24/24	31.04	0.07										
	4	20/24	33.33	0.12										
	0.8	18/24	35.15	0.14										
	0.16	7/24	36.20	0.17										
	0.032	3/24	36.60	0.27										
	0	0/24	0.00	0.00										

2	62500	8/8	21.03	0.07	105%	0.998	0.2	0.3	0.6	0.8	1.2	2.1	4	0
	12500	8/8	23.23	0.07										
	2500	8/8	25.54	0.07										
	500	8/8	27.88	0.04										
	100	24/24	30.03	0.06										
	20	24/24	31.16	0.06										
	4	24/24	33.69	0.16										
	0.8	19/24	35.37	0.17										
	0.16	6/24	36.72	0.13										
	0.032	3/24	36.45	0.10										
	0	0/24	#DIV/0!	#DIV/0!										
3	62500	8/8	20.19	0.15	97%	0.9983	0.2	0.3	0.6	0.8	1.2	2.2	4	0
	12500	8/8	22.55	0.22										
	2500	8/8	24.81	0.16										
	500	8/8	27.19	0.21										
	100	17/24	29.05	1.55										
	20	24/24	30.83	0.34										
	4	24/24	33.34	0.77										
	0.8	18/24	35.52	0.90										
	0.16	8/24	35.88	1.05										
	0.032	1/24	36.73	#DIV/0!										
	0	1/24	42.60	#DIV/0!										

Additional table 7. Raw data and calculated sensitivity parameters for eLIPI1 sensitivity validation using synthetic gDNA (gBlocks®) dilutions. LL, lower limit; LOB, Limit of blank; LOD, limit of detection; LOQ, limit of quantification; $LOQ_{continuous}$, continuous LOQ; UL, upper limit; rxn, reaction

Month	Total Copies/rxn	Frequency	Average	Standard Error	Efficiency	R^2	LOD LL (copies/rxn)	LOD (copies/rxn)	LOD UL (copies/rxn)	LOQ LL (copies/rxn)	LOQ (copies/rxn)	LOQ UL (copies/rxn)	$LOQ_{continuous}$ (copies/rxn)	LOB
0	62500	8/8	20.72	0.07	107%	0.996	0.2	0.2	0.4	0.6	0.9	1.5	4	0
	12500	8/8	23.04	0.10										
	2500	8/8	25.26	0.18										
	500	8/8	27.46	0.31										
	100	24/24	29.00	0.65										
	20	24/24	32.22	0.07										
	4	24/24	34.06	0.13										
	0.8	18/24	35.64	0.16										
	0.16	6/24	37.95	0.12										
	0.032	0/24	0.00	0.00										
	0	0/24	0.00	0.00										
1	62500	8/8	20.57	0.05	99%	0.9998	0.2	0.2	0.4	0.6	0.9	1.6	4	0
	12500	8/8	22.84	0.03										
	2500	8/8	25.27	0.02										
	500	8/8	27.48	0.05										
	100	24/24	30.02	0.10										
	20	24/24	32.34	0.10										
	4	24/24	34.51	0.11										
	0.8	19/24	37.00	0.18										
	0.16	4/24	37.40	0.05										
	0.032	0/24	0.00	0.00										
	0	0/24	0.00	0.00										

2	62500	8/8	21.42	0.05	107%	0.9983	0.2	0.2	0.4	0.6	0.8	1.4	4	0
	12500	8/8	23.78	0.05										
	2500	8/8	26.24	0.05										
	500	8/8	28.38	0.03										
	100	24/24	30.59	0.08										
	20	24/24	32.33	0.07										
	4	24/24	34.90	0.14										
	0.8	18/24	36.75	0.18										
	0.16	6/24	37.03	0.13										
	0.032	1/24	36.54	#DIV/0!										
	0	0/24	#DIV/0!	#DIV/0!										
3	62500	8/8	20.47	0.14	102%	0.9998	0.2	0.3	0.5	0.7	1	1.7	4	0
	12500	8/8	22.84	0.21										
	2500	8/8	25.12	0.14										
	500	8/8	27.46	0.18										
	100	24/24	29.78	0.26										
	20	24/24	31.97	0.34										
	4	24/24	34.18	0.77										
	0.8	15/24	36.68	1.09										
	0.16	5/24	37.12	0.42										
	0.032	3/24	36.50	0.71										
	0	1/24	48.46	#DIV/0!										

Additional table 8. Raw data and calculated sensitivity parameters for eONMY5 sensitivity validation using synthetic gDNA (gBlocks®) dilutions. LL, lower limit; LOB, Limit of blank; LOD, limit of detection; LOQ, limit of quantification; $LOQ_{continuous}$, continuous LOQ; UL, upper limit; rxn, reaction

Month	Total Copies/rxn	Frequency	Average	Standard Error	Efficiency	R^2	LOD LL (copies/rxn)	LOD (copies/rxn)	LOD UL (copies/rxn)	LOQ LL (copies/rxn)	LOQ (copies/rxn)	LOQ UL (copies/rxn)	$LOQ_{continuous}$ (copies/rxn)	LOB
0	62500	8/8	20.83	0.08	103%	0.9997	0.3	0.4	0.7	0.9	1.4	2.7	4	0
	12500	8/8	23.11	0.06										
	2500	8/8	25.50	0.04										
	500	8/8	27.76	0.06										
	100	24/24	30.00	0.07										
	20	24/24	32.37	0.18										
	4	24/24	34.43	0.15										
	0.8	15/24	36.71	0.17										
	0.16	1/24	37.26	0.00										
	0.032	1/24	36.16	0.00										
	0	0/24	0.00	0.00										
1	62500	8/8	20.69	0.04	89%	0.998	0.2	0.3	0.6	0.8	1.2	2.1	4	0
	12500	8/8	23.07875	0.05										
	2500	8/8	25.32	0.06										
	500	8/8	27.8725	0.04										
	100	24/24	30.562916	0.14										
	20	24/24	32.850833	0.16										
	4	24/24	36.03125	0.33										
	0.8	15/24	35.458666	1.45										
	0.16	4/24	38.9875	0.82										

	0.032	1/24	37.06	0.00									
	0	0/24	0	0.00									
2	62500	8/8	21.11	0.03	103%	0.9993	0.3	0.4	0.7	1	1.4	2.8	4	0
	12500	8/8	23.37	0.04									
	2500	8/8	25.79	0.05									
	500	8/8	28.21	0.04									
	100	24/24	30.43	0.06									
	20	24/24	32.51	0.09									
	4	24/24	34.70	0.14									
	0.8	14/24	36.44	0.23									
	0.16	2/24	38.87	0.41									
	0.032	1/24	37.41	#DIV/0!									
	0	0/24	#DIV/0!	#DIV/0!									
3	62500	8/8	20.72	0.07	99%	0.9993	0.2	0.3	0.5	0.7	1.1	1.9	4	0
	12500	8/8	22.95	0.08									
	2500	8/8	25.26	0.06									
	500	8/8	27.78	0.09									
	100	24/24	30.33	0.28									
	20	24/24	32.32	0.09									
	4	24/24	34.68	0.17									
	0.8	16/24	37.13	0.34									
	0.16	4/24	39.39	1.03									
	0.032	1/24	42.82	#DIV/0!									
	0	1/24	40.74	#DIV/0!									
